# Therapeutic plasma exchange in acute liver failure: Unravelling its efficacy and impact

**DOI:** 10.1016/j.htct.2026.106268

**Published:** 2026-04-17

**Authors:** Sonal Sonu, Rajesh Kumar, Prince Patel, Gulinder Singh, Amanpreet Kaur, Sonia Gupta, Deepika Aggarwal

**Affiliations:** Department of Immunohematology and Blood Transfusion, Dayanand Medical College and Hospital, India

**Keywords:** Acute liver failure, Therapeutic plasma exchange, Model for end-stage liver disease (MELD) scores

## Abstract

**Background:**

Acute liver failure presents a significant medical challenge due to its rapid onset, progression, and high mortality rates, often necessitating liver transplantation. Therapeutic plasma exchange has emerged as a potential intervention to modulate the underlying pathophysiological mechanisms and to reduce the need for immediate transplantation.

**Methods:**

This retrospective monocentric study was conducted at a tertiary referral hospital from June 2023 to March 2024 to assess the efficacy of therapeutic plasma exchange in acute liver failure patients. Clinical parameters, including biochemical markers, coagulation status, and Model for End-Stage Liver Disease scores, were analyzed pre- and post-plasma exchange. Additionally, hospitalization duration and adverse events associated with the procedure were documented.

**Results:**

Twenty-five patients with acute liver failure underwent therapeutic plasma exchange resulting in significant improvements in biochemical markers, notably reductions in bilirubin, aspartate transaminase, and alanine transaminase levels (p-value <0.001 for all). Coagulation parameters and Model for End-Stage Liver Disease scores exhibited favorable trends, particularly among discharged patients. Hospitalization duration varied across patient outcomes, indicating the impact of the procedure on clinical course. Adverse reactions were reported but remained manageable, with no severe complications observed.

**Conclusion:**

Therapeutic plasma exchange demonstrates promising efficacy in stabilizing acute liver failure patients, improving biochemical markers, and potentially mitigating the urgency for immediate transplantation. Despite challenges such as donor scarcity and adverse events, the safety profile remains favorable, suggesting the potential of plasma exchange as a valuable adjunct in acute liver failure management. Further research and clinical scrutiny are warranted to fully ascertain the role of therapeutic plasma exchange in optimizing outcomes.

## Introduction

In the complex landscape of acute liver failure (ALF), where every moment is critical, the liver's pivotal role in sustaining life comes sharply into focus. Responsible for synthesizing vital proteins, producing coagulation factors, and detoxifying the bloodstream, dysfunction in ALF precipitates a cascade of challenges, including coagulopathy, toxin accumulation, and profound prognostic uncertainty [1].

ALF is characterized by a rapid clinical deterioration, marked by the emergence of encephalopathy following jaundice and coagulopathy, particularly in patients without preexisting chronic liver disease. The etiology of ALF demonstrates regional variations. Acetaminophen is the dominant etiology of ALF in the developed world; viral and drug-induced etiologies are frequently encountered in developing countries [2]. The immune dysfunction in ALF patients leads to the development of infections, cerebral edema, and multiorgan dysfunction syndrome, necessitating prompt diagnosis and expedited management [3, 4, 5].

Despite advances in medical science, the mortality rate of ALF remains dauntingly high, reaching 60–80 % with conservative management alone. Liver transplantation, the gold standard treatment, faces formidable obstacles, chiefly the scarcity of viable donors [6]. In this backdrop, the quest for innovative therapies to bridge the gap between diagnosis and transplantation becomes imperative.

Therapeutic plasma exchange (TPE) is a cornerstone of liver support systems, heralded for its ability to target the underlying pathophysiology of ALF. Endorsed by the American Society for Apheresis (ASFA), TPE offers a multifaceted approach, removing toxins, replenishing coagulation factors, and restoring fluid balance. Its utility is particularly evident in fulminant hepatic failure, where it serves as a vital bridge to transplantation. Current guidelines give a strong grade 1A/I recommendation for high-volume (HV)-TPE but only a weak grade 2B/III recommendation for using any non-HV-TPE in ALF, given the lack of robust data for a low-volume (LV)-TPE approach [7].

This study provides a comprehensive evaluation of the efficacy of TPE in critically ill patients with liver disease. By elucidating the impact of TPE on clinical outcomes, this research aims to refine management strategies for liver failure and optimize therapeutic protocols for this high-acuity population.

## Material and methods

### Study population

This retrospective, monocentric investigation was conducted at a tertiary referral hospital over a period spanning from June 2023 to March 2024. Patients presenting with acute chronic liver failure (ACLF) and those with secondary ALF arising from concurrent critical conditions such as septic shock or cardiogenic shock were deliberately excluded. This study specifically focused on patients meeting the criteria for ALF, as outlined by the Asian Pacific Association for the Study of the Liver (APASL). ALF is a critical medical emergency characterized by severe hepatic injury resulting in coagulopathy, typically defined by an International Normalized Ratio (INR) ≥1.5, and the presence of hepatic encephalopathy in a patient without pre-existing liver disease. This syndrome is clinically recognized when the duration of illness is <26 weeks; hyperacute and acute presentations typically occur within one to four weeks of symptom onset [8]. The etiologies of ALF in this study included hepatitis A (72 %; *n* = 18), hepatitis E (24 %; *n* = 6), and drug-induced liver injury (4 %; *n* = 1). Yellow fever was not reported, as it is non-endemic in the region. The institute’s ethics committee approved the research protocol.

### Intervention - therapeutic plasma exchange

Vascular access was established via venous insertion of a 12-French triple-lumen hemodialysis catheter. TPE procedures were performed by a transfusion medicine specialist with a Spectra Optia Apheresis System and MCS^+®^ 9000 Mobile Collection System. Each TPE session involved the exchange of approximately 1–1.5 plasma volume, guided by clinical indications and the treating clinician's requisition. TPE was performed daily following the diagnosis of ALF until clinical recovery or liver transplantation, leading to a variable number of interventions per patient (generally 3–5 sessions). The standardized replacement fluid dosage comprised 10–12 units of fresh frozen plasma (FFP) and saline.

The estimated plasma volume for each patient was calculated using the body weight and hematocrit in the formula to prescribe TPE as follows:

Estimatedplasmavolume=(0.65xbodyweight)x(1−hematocrit)

[9]

Modifications were made in the calculation of the blood volume based on gender and age as follows:

Gender−basedtotalbloodvolume(TBV)=idealbodyweightx75mL/kg(male)or65mL/kg(female)

Age−basedmodifiedTBV=idealbodyweightx70mL/kg(<65yearsold)or60mL/kg(≥65yearsold)

[10]

Each TPE session lasted for 3–4 h. An acid-citrate-dextrose (ACD) anticoagulant solution (1:12 vol ratio anticoagulant to whole blood) was used during the procedure. Rates of citrate flow were meticulously adjusted to keep post-filter ionized calcium concentration in a target range of 0.5–0.6 mmol/L, and caution was taken while supplementing calcium intravenously in the form of calcium gluconate in normal saline (10 mL in 10 % w/v) over 20–30 min with an infusion pump, for every 1000–1200 mL of replacement fluid to avoid citrate toxicity. Calcium concentrations were monitored by venous blood gas (VBG), and calcium gluconate was administered as required. Platelet count, prothrombin time (PT), INR, and liver function tests (total bilirubin, direct bilirubin, indirect bilirubin, aspartate transaminase [AST], alanine transaminase [ALT] and serum creatinine) were used as pre- and post-procedure laboratory assessments. Outcomes were also assessed through the Model for End-Stage Liver Disease (MELD) score and clinical status.

The laboratory parameters and MELD scores were expressed as mean ± SD with analysis employing the paired *t*-test and one-way ANOVA. GraphPad Prism (Trail Version GraphPad Software) and IBM SPSS Statistics (Version 25.0, IBM Corp., Armonk, New York) were used for data analysis and graph generation. The result was considered statistically significant when the two-tailed p-value was <0.05.

## Results

During the study period, 25 patients were diagnosed with ALF and underwent TPE. The cohort had a mean age of 21.48 years, with a marked male predominance (84 %; *n* = 21) compared to females (16 %; *n* = 4). At hospital admission, Grade II and III hepatic encephalopathy (HE) was present in 48 % of cases. Each of the procedures was conducted daily (3–5 procedures; mean of 3.1 procedures). The median treatment time was 240 min (210–260 min). An average of 10–12 units of FFP were used with the average exchanged plasma volume being 3.2 ± 0.8 liters per session. In addition, 56 % of patients required continuous renal replacement therapy (CRRT) due to acute kidney injury at inclusion. Ammonia levels, an important indicator that determines the management of HE, showed a significant decrease from 92.45 µmol/L (± 45.23) pre-procedure to 45.62 µmol/L (± 24.78) post-procedure (p-value = 0.002), which was suggestive of a positive impact of the procedure on hepatic function and metabolic stability. CRRT was temporarily stopped during TPE sessions in patients with acute kidney injury.

**Figure 1:**
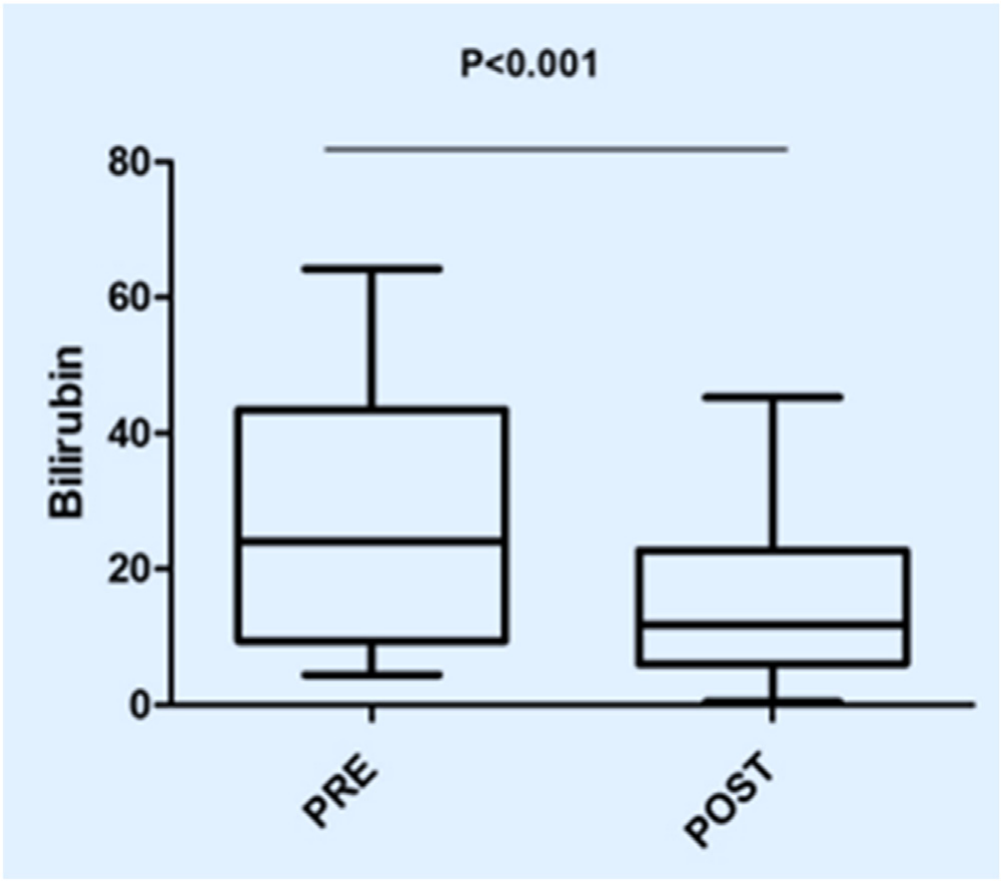
Impact of therapeutic plasma exchange on bilirubin before and after therapeutic plasma exchange.

**Figure 2:**
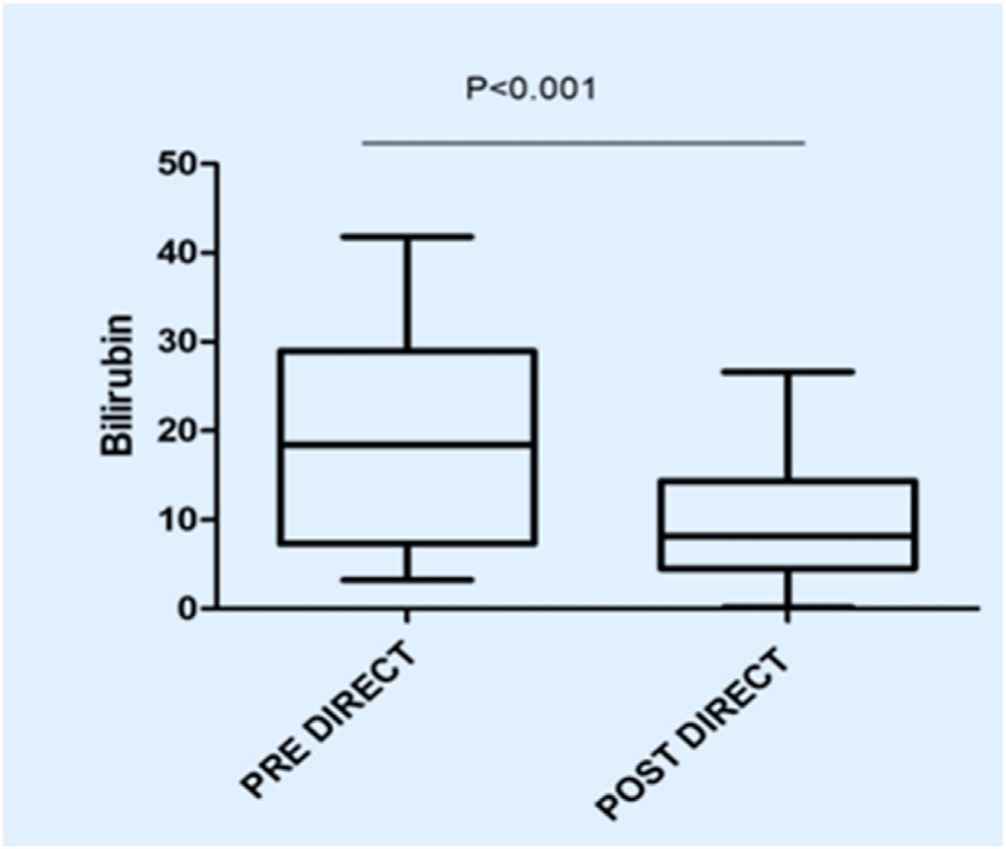
Direct bilirubin levels before and after therapeutic plasma exchange.

**Figure 3:**
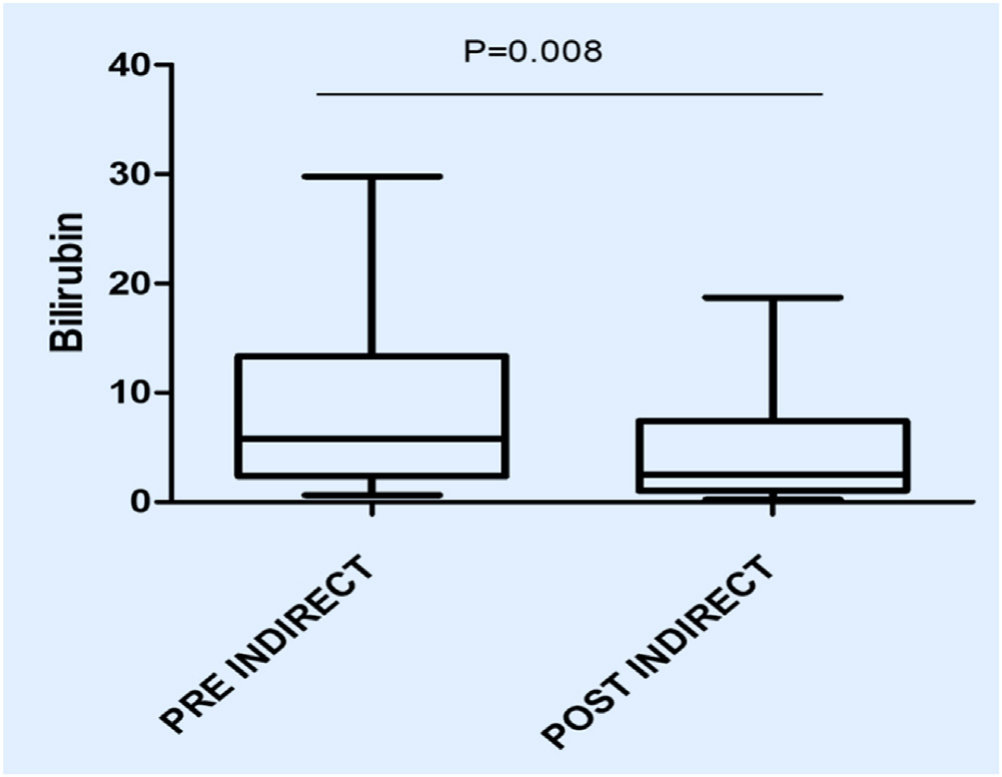
Indirect bilirubin levels before and after therapeutic plasma exchange.

**Figure 4:**
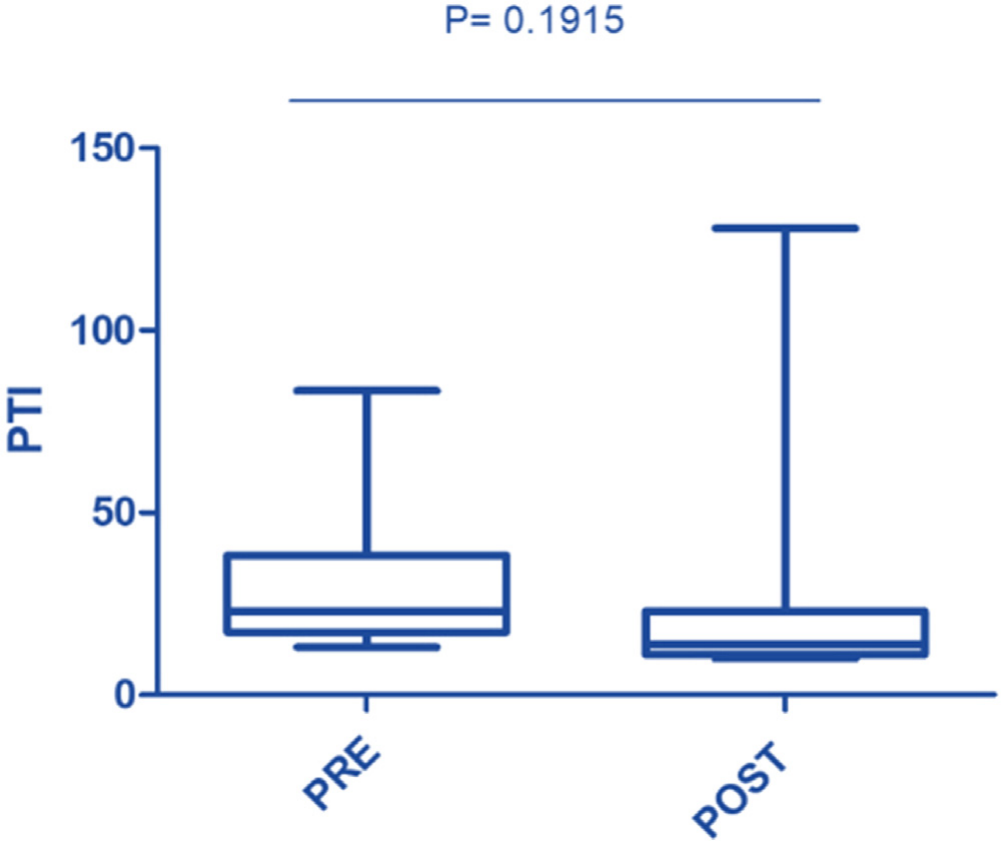
Prothrombin Time Index (PTI) before and after therapeutic plasma exchange.

**Figure 5:**
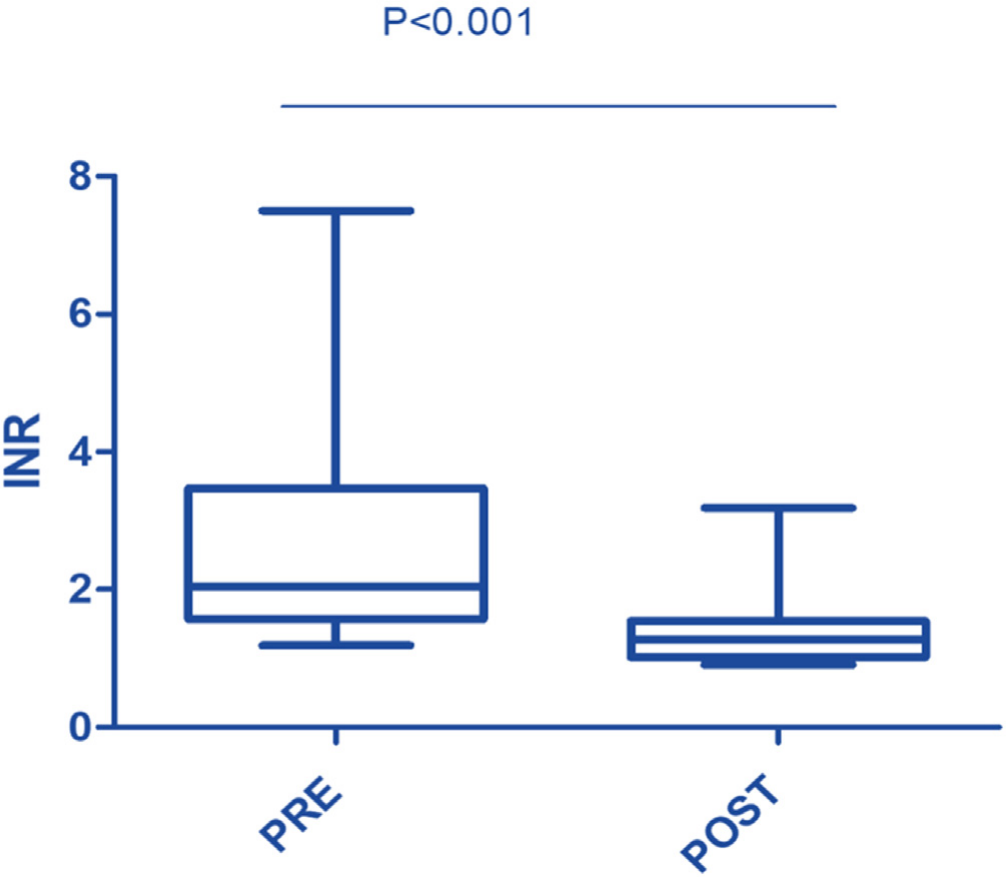
International Normalized Ratio (INR) levels before and after therapeutic plasma exchange.

**Figure 6:**
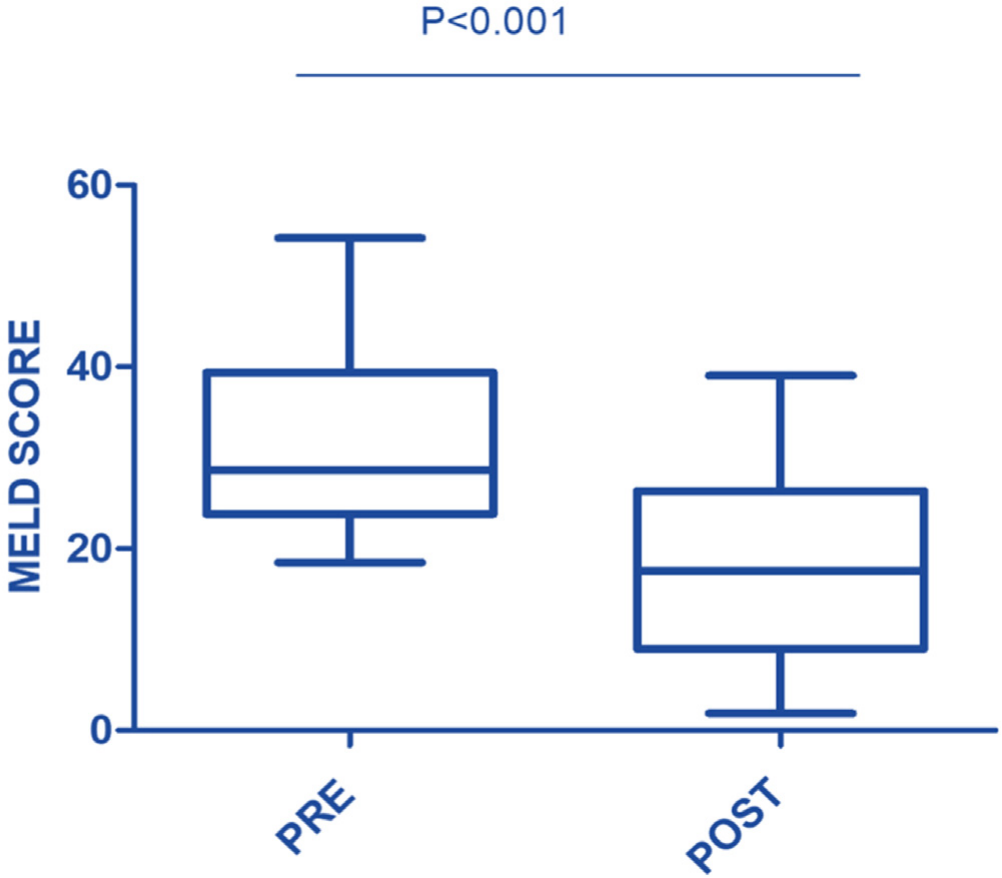
Model for End-Stage Liver Disease (MELD) score before and after therapeutic plasma exchange.

**Figure 7:**
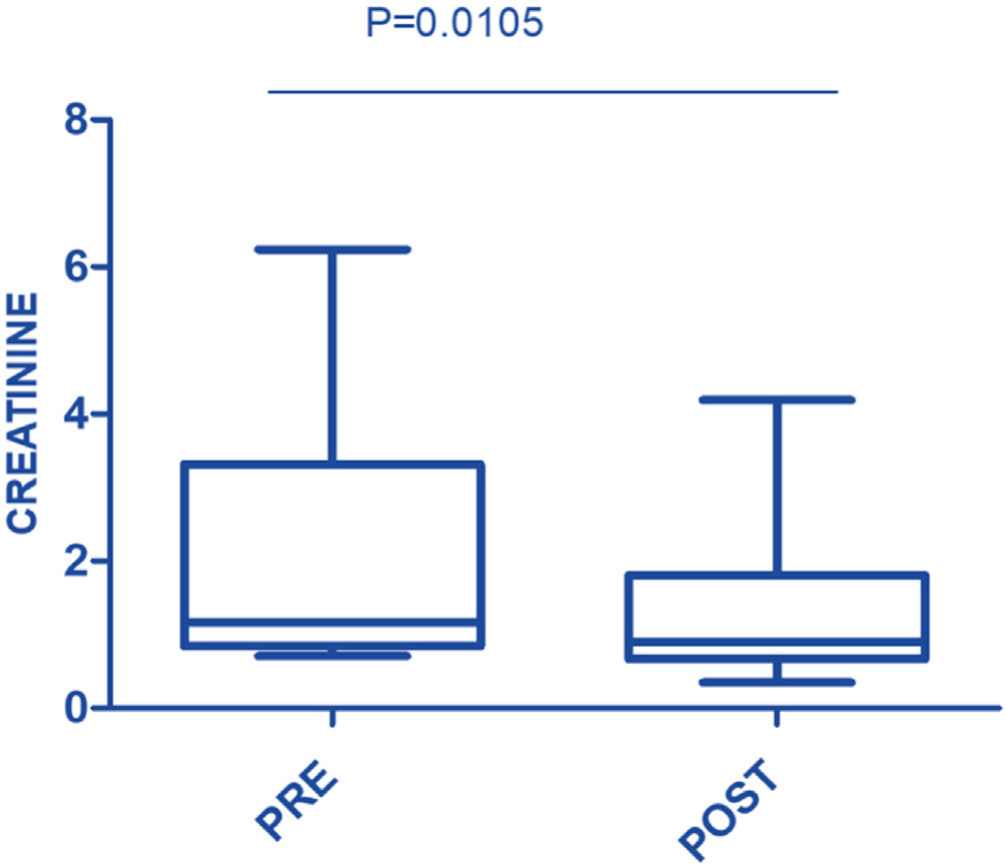
serum creatinine levels before and after therapeutic plasma exchange.

**Figure 8:**
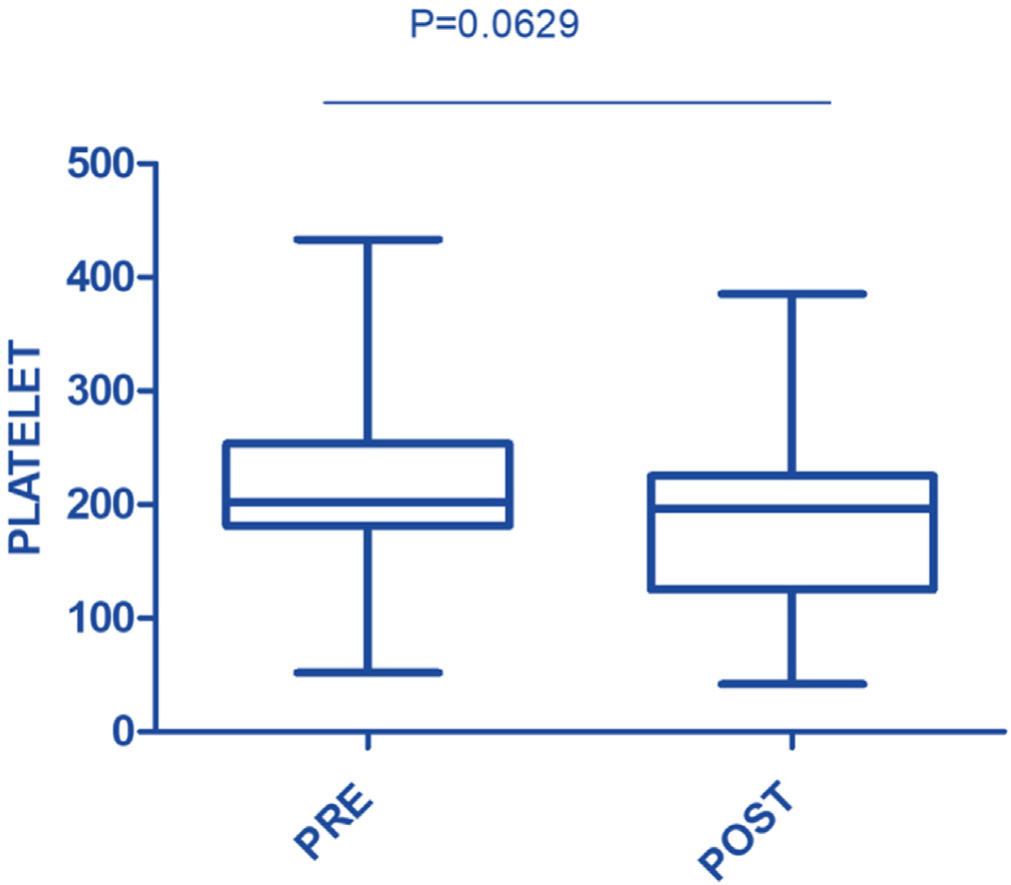
Platelet count in response to therapeutic plasma exchange.

In this study, the procedure improved several biochemical parameters. The mean total bilirubin levels dropped significantly from a baseline of 27.48 ± 19.82 mg/dL to 14.64 ± 11.75 mg/dL after the procedure (p-value = 0.001) (Figure 1). Likewise, direct bilirubin levels significantly dropped from 18.85 ± 12.43 mg/dL to 9.88 ± 7.38 mg/dL after the intervention (p-value <0.001) (Figure 2). At the same time, indirect bilirubin levels were also significantly reduced from 8.33 ± 7.74 mg/dL to 4.76 ± 5.14 mg/dL post-procedure (p-value = 0.008) (Figure 3). Reductions in AST and ALT levels were also seen, with post-procedure AST declining from 2243.60 ± 1830.58 IV/L to 199.12 ± 165.49 IV/L (p-value <0.001) and ALT dropping from 2521.00 ± 1739.22 IV/L to 261.08 ± 202.19 IV/L (p-value <0.001) (Figure 4). These results further confirm the potential of this intervention to modulate biochemical markers of liver function. The patients had a mean INR of 2.62 ± 1.55 before TPE, which was significantly decreased to 1.47 ± 0.63 after TPE, indicating a better coagulation profile (Figure 5). Moreover the Prothrombin time reduced from 29.19 ± 17.23 to21.55 ± 23.79 (p-value = 0.32) (Figure 6). The creatinine level dropped from 2.22 ± 184 to 1.36 ± 1.62 mg/dL (p-value = 0.47) (Figure 7) There was a reversible decrease in platelet count during TPE, from 217.04 ± 77.14 to 180.96 ± 78.24 (p-value = 0.063) (Figure 8). Cohen’s *d* statistics were calculated to quantify the effect sizes of the differences observed between pre-TPE and post-TPE measurements. The analysis yielded *d* values ranging from 0.32 (95 % confidence interval [95 % CI]: 0.15–0.49) to 1.43 (95 % CI: 1.22–1.63), representing a spectrum from small to large effect sizes across the majority of analyzed parameters (Table 1).

**Table 1:** Comparative analysis of laboratory parameters before and after therapeutic plasma exchange (TPE).

**No.**	**Parameter**	**Before TPE** **mean ± SD**	**After TPE** **mean ± SD**	**t-value**	**p-value**	**Cohen's d**
1.	Ammonia Level µmol/L)	92.45 ± 45.23	45.62 ± 24.78	1.817	0.002	1.282
2.	Total Bilirubin (mg/dL)	27.48 ± 19.82	14.64 ± 11.75	4.019	0.001	0.59
3.	Direct Bilirubin (mg/dL)	18.85 ± 12.43	9.88 ± 7.38	4.399	<0.001	0.73
4.	Indirect Bilirubin (mg/dL)	8.33 ± 7.74	4.76 ± 5.14	2.867	0.008	0.67
5.	AST (IV/L) (SGOT)	2243.60 ± 1830.58	199.12 ± 165.49	5.552	<0.001	0.91
6.	ALT (IV/L) (SGPT)	2521.00 ± 1739.22	261.08 ± 202.19	6.310	<0.001	1.43
7.	Platelet Count (/µL)	217.04 ± 77.14	180.96 ± 78.24	1.950	0.063	0.44
8.	Prothrombin time (S)	29.19 ± 17.23	21.55 ± 23.79	1.344	0.191	0.32
9.	International Normalized Ratio	2.62 ± 1.55	1.47 ± 0.63	3.843	0.001	0.72
10.	Serum Creatinine (mg/dL)	2.22 ± 1.84	1.36 ± 1.62	2.774	0.110	0.47
11.	MELD score	31.69 ± 9.67	17.82 ± 10.38	6.536	<0.001	0.89

AST: aspartate transaminase; SGOT: Serum glutamic-oxaloacetic transaminase; ALT: alanine transaminase; SGPT: Serum glutamic-pyruvic transaminase; SD: Standard deviation.

Patient outcomes were compared with hospitalization durations. According to the findings, there is a large difference in the length of stay, showing the shortest was mortality with 8.86 days, significantly diverging from other groups (*f* = 18.705; p-value <0.001). Discharge against medical advice (DAMA) patients experienced a moderate stay of 18.27 days, while those discharged stayed the longest at 56.00 days (Table 2). The results highlight the impact of patient outcomes and how substantial savings can be achieved if care pathways are tailor-made to enhance both resource utilization and patient outcomes.

**Table 2:** Comparison of hospital stay duration by outcome.

**Outcome**	**N**	**Mean Stay**	**SD**	**F-value**	**P-value**
Mortality	7	8.86	6.122	18.705	<0.001
DAMA	11	18.27	10.355		
Discharge	7	56.00	25.794		
**Total**	**25**	**26.20**	**24.411**		

SD: Standard deviation; DAMA: Discharge against medical advice.

In cases where patients succumbed to their conditions, their MELD score before TPE was 36.683 ± 11.124, decreasing to 28.898 ± 8.766 post-TPE, although there was no significant difference between them (*t* = 2.104; p-value = 0.080). Conversely, a statistically significant reduction (*t* = 5.868; p-value = 0.001) in MELD scores was observed among patients who were discharged. In this subgroup, MELD scores decreased substantially from 33.767 ± 10.912 to 11.871 ± 8.016 following TPE demonstrating the clinical utility of TPE in improving the prognosis for surviving patients. An interesting observation was seen in DAMA patients. Despite exhibiting a significant reduction in MELD scores post-TPE, from 27.189 ± 5.998 to 15.822 ± 9.325 (*t* = 10; p-value = 0.001), these patients faced challenges in obtaining further treatment, likely due to financial constraints (Table 3). This observation highlights a critical barrier to healthcare access, wherein patients who may have been demonstrating improvement were compelled to discontinue treatment prematurely due to financial limitations. Moreover, the statistically significant reduction in MELD scores among patients discharged post-TPE highlights the pronounced positive impact of TPE on disease progression and patient outcomes.

**Table 3:** Comparison of Model for End-Stage Liver Disease (MELD) scores before and after therapeutic plasma exchange (TPE) across different outcomes.

**Outcome**	**MELD Score**			
	**Before TPE** **mean ± SD**	**After TPE** **mean ± SD**	**t-value**	**p-value**
Mortality	36.683 ± 11.124	28.898 ± 8.766	2.104	0.080
Discharged	33.767 ± 10.912	11.871 ± 8.016	5.868	0.001
DAMA	27.189 ± 5.998	15.822 ± 9.325	10	0.001

SD: Standard deviation; DAMA: Discharge against medical advice.

Results show that the use of TPE was viable and well tolerated, facilitating its integration into routine care. However, adverse reactions were described with itching (15 %), urticaria (14 %), and hypocalcemia (20 %) being the most common occurring. However, no major adverse events, such as anaphylactic reactions or transfusion-related acute lung injury (TRALI), were found. Citrate was effectively used as an anticoagulant in all patients, with no significant electrolyte imbalances or acid-base disturbances recorded.

## Discussion

The clinical benefit of TPE in patients with ALF was evaluated by analyzing specific biochemical parameters and determining the prognostic utility of the procedure within the current cohort. The prevalence of Grades II and III hepatic encephalopathy (HE) in 48 % of patients upon admission is consistent with the clinical presentations documented by Bernal et al. [11], highlighting the severe neurological impact of ALF. Acute kidney injury necessitating CRRT was present in 56 % of patients at inclusion. In these patients, CRRT was temporarily suspended during TPE sessions. This is in agreement with the management techniques described by Céline Monard et al. [12]. Interruptions of extracorporeal treatments may be needed in the CRRT schedule to avoid complications. One of the significant clinical study findings is a considerable reduction in the serum ammonia level after TPE, suggesting a beneficial effect on hepatic function and metabolic stability, which are critical in the control of HE [13,14]. This highlights the multi-factorial advantages of TPE in ALF, managing the liver and neurological sequelae of ALF. Consistent with the findings of Singer et al. [15], the present study demonstrated significant reductions in bilirubin and improvements in coagulation parameters (INR). Singer et al. emphasized the role of TPE in enhancing liver function by effectively removing hepatic toxins and improving coagulation status. The same is true of the present results, which confirmed that TPE can effectively reach these clinical objectives.

MELD score analysis showed a significant drop in the scores of discharged patients post-TPE (from 33.767–11.871), emphasizing the role of TPE in reducing disease severity. This was comparable to the findings reported by Freeman et al. [16], who observed a 55 % survival rate in post-TPE liver failure patients. The lack of reduction in MELD scores among the deceased (36.683–28.898) indicates that although TPE may improve biochemical parameters, this may not translate to significant survival advantages in critically ill patients. The marked fall in MELDs in DAMA patients (27.189–15.822) is an important impediment to access to health care for financial reasons; these are similar observations to those of Lee et al. [17]. This emphasizes the importance of policy interventions to improve treatment access and adherence. Those who died had spent an average of 8.86 days hospitalized, whereas the average length of stay for those discharged was 56.00 days. The importance of early intervention and persistence of care in managing patient outcomes is an overarching theme highlighted in these studies and research by Shalimar et al. [18].

This study did not directly measure the rate of transplantation. Given the critical role of liver transplantation in ALF management, it is important to acknowledge that at the time of the current study, liver transplant services were not available at the study institution, and eligible patients were referred to other centers for transplantation. However, the findings of this research suggest that TPE may offer a therapeutic window for patient stabilization, potentially reducing mortality while awaiting transplantation. Nevertheless, major improvements in biochemical markers and clinical status raise the chance that TPE could allow delayed transplantation in the very short term by stabilizing patients and allow more time to obtain an appropriate donor. The same result was also seen in the study of Larsen et al., in which TPE was associated with better liver function tests and survival in patients with ALF. Their study showed that early TPE was effective in significantly reducing the severity of liver injury and acted as an important bridge to transplantation when required [19].

No severe side effects, like anaphylactic reactions or TRALI, were reported, consistent with the safety profiles [20]. The efficient use of citrate as an anticoagulant without developing major electrolyte abnormalities or acid-base balance appears to align with other observations [21]. Moreover, patients demonstrated significant improvements in patient-reported outcomes regarding quality of life, with the magnitude of benefit exceeding initial clinical expectations. This could indicate that the benefits of TPE extend beyond just biochemical corrections and result in an overall improvement in patient well-being and recovery.

## Conclusions

Our data highlight the potential use of TPE as an effective adjunct in ALF. By optimizing biochemical markers and clinical outcomes, TPE facilitates patient stabilization and improves prognosis; this therapeutic strategy serves as a critical bridge to liver transplantation or, ideally, reduces the necessity for an emergency surgical intervention. In the context of ALF management, when the bridge from diagnosis to transplantation is too long, TPE certainly rises as a transitory bridge that may represent potential good patient outcomes and optimal health strategies. Though it has the potential to treat ALF, more studies and clinical judgment support are necessary to exploit the power of TPE in the comprehensive treatment of ALF.

## Credit authorship contribution statement

S.S. and R.K. contributed to conceptualization, methodology, analysis, and supervision. They led the writing and editing. P.P. served as the statistician, handling data review and validation. G.S. managed software, validation, and analysis. A.K. focused on visualization and assisted with writing and editing. S.G. managed resources and administration. D.A. handled data curation and supported writing and editing.

## Data availability

The data that support the findings of this study are available from the corresponding author upon reasonable request.

## Conflicts of interest

The authors declare no conflict of interest.
